# Release of Gentamicin and Vancomycin from Preformed Spacers in Infected Total Hip Arthroplasties: Measurement of Concentrations and Inhibitory Activity in Patients' Drainage Fluids and Serum

**DOI:** 10.1155/2013/752184

**Published:** 2013-09-22

**Authors:** Dario Regis, Andrea Sandri, Elena Samaila, Anna Benini, Manuel Bondi, Bruno Magnan

**Affiliations:** ^1^Department of Orthopaedic and Trauma Surgery, Integrated University Hospital, 37126 Verona, Italy; ^2^Department of Public Health and Community Medicine, Pharmacology Section, University of Verona, 37134 Verona, Italy

## Abstract

Gentamicin (G) and vancomycin (V) concentrations in drainage fluids obtained from patients during the first 24 hours after implantation of antibiotic-loaded polymethylmethacrylate (PMMA) spacers in two-stage revision of infected total hip arthroplasty were studied. The inhibitory activity of drainage fluids against different multiresistant clinical isolates was investigated as well. Seven hips were treated by implantation of industrial G-loaded spacers. Vancomycin was added by manually mixing with PMMA bone cement. Serum and drainage fluid samples were collected 1, 4, and 24 hours after spacer implantation. Antibiotics concentrations and drains bactericidal titer of combination were determined against multiresistant staphylococcal strains. The release of G and V from PMMA cement at the site of infection was prompt and effective. Serum levels were below the limit of detection. The local release kinetics of G and V from PMMA cement was similar, exerting a pronounced, combined inhibitory effect in the implant site. The inhibitory activity of drainage fluids showed substantial intersubject variability related to antibiotic concentrations and differed according to the pathogens tested. Gentamicin and vancomycin were released from temporary hip spacers at bactericidal concentrations, and their use in combination exerted strong inhibition against methicillin-resistant *S. aureus* and Coagulase Negative Staphylococci strains.

## 1. Introduction

Polymethylmethacrylate (PMMA) cements preloaded with antibiotics, mainly gentamicin (G), are used in some cases for prophylaxis but especially for the surgical revision of prosthetic infections [1]. Frequent microorganisms isolated from joint fluid or periprosthetic tissue are the Coagulase Negative Staphylococci (CoNS), *S. aureus* and most commonly *S. epidermidis*, and *Streptococcus haemolyticus* [2]. Currently, the routinely used methods of culturing are likely to detect in most, not all, cases the pathogens possibly involved in infection of a total hip arthroplasty (THA) [3]. Some difficult-to-treat bacteria, such as methicillin-resistant *S. aureus* (MRSA), methicillin-resistant CoNS, enterococci, and *Pseudomonas aeruginosa* present much greater failure risks. In a number of cases *S. aureus* infection is the significant factor associated with treatment failure, along with retained prosthesis and treatment with inappropriate antibiotics [4]. A two-stage revision of an infected arthroplasty with antibiotic-loaded spacer implantation is considered an effective procedure for these infections [2, 5]. 

Because of the increasing resistance of staphylococci to gentamicin, surgeons commonly add antibiotics to bone cement directly in the operating room according to microorganism susceptibility. Vancomycin (V) is frequently utilized because of its antimicrobial activity against MRSA and other Gram-positive cocci and anaerobes, such as propionibacteria.

This drug delivery system offers the advantage of local release of high antibiotic concentrations, which considerably exceed those obtained after systemic administration. 

Combining two antibiotics in bone cement is common in clinical practice. As the effect of mixing on elution characteristics is still debated, only limited data are available on antibiotic release *in vivo* from prosthetic devices after implantation [6–8], as well as after removal [9, 10]. Moreover, the wide variability of the results makes it difficult to compare studies and draw general conclusions. The properties of various bone cements, the preparation, type and concentrations of different antibiotics mixed with PMMA, the pathogens involved, and patient characteristics are all factors contributing to the clinical outcome and should be taken into account in the final evaluation of treatments. We previously observed that gentamicin and vancomycin were still present in explanted spacers after 3 to 9 months of permanence in situ; the residual drug concentrations showed great variability [11]. 

Open questions are related to the concentrations of antibiotics at the implantation site and to the duration of their effective inhibitory activity.

The aims of the present study were (1) to measure the concentrations of antibiotics present at the infection site in the first few hours after implanting the hip preformed spacer; (2) to evaluate if antibiotics are released in large amounts consistent with the results of *in vitro* experiments; (3) and finally to assess the antimicrobial activity of drainage fluids against multiresistant microorganisms.

To the best of our knowledge, this is the first study which details the antibiotic release from industrially manufactured temporary spacers in infected hip arthroplasties.

## 2. Materials and Methods

From January 2004 to September 2005, 7 patients who received preformed spacers for two-stage revision of a THA were investigated. There were 4 male and 3 female patients, whose age ranged between 51 and 78 years (average, 65.6 years) at the time of implantation. All THAs were performed for osteoarthritis. Diagnosis was suspected on the basis of clinical findings (persistent pain or recurrence 3–5 years later, presence of a secreting fistula, swelling, erythema, local warmth, and restricted range of motion) and of the ESR and the CRP (which were always elevated) [12]. Standard X-ray and scintigraphy with labeled leukocytes were performed in all the patients [12–16]. Intraoperative biopsy of bone and soft tissue was always carried out, and the Feldman and the Athanasou criteria were used to define infection [17, 18]. The management of infection included removal of the prosthesis and insertion of a preformed antibiotic-loaded PMMA spacer (Tecres S.p.A., Sommacampagna, Verona, Italy).

The hip preformed spacer, Spacer-G, has a structure in stainless steel AISI 316ESR, and it is available in 3 different diameters of the head (46, 54, and 60 mm) and 2 lengths of the stem (153 and 270 mm). The gentamicin concentration is 2.5%. Currently, it is also available with flat rod (Flat Stem Spacer-G) and industrially supplemented with vancomycin at concentration of 2.5% (Vancogenx Hip-Space).

**Figure 1:**
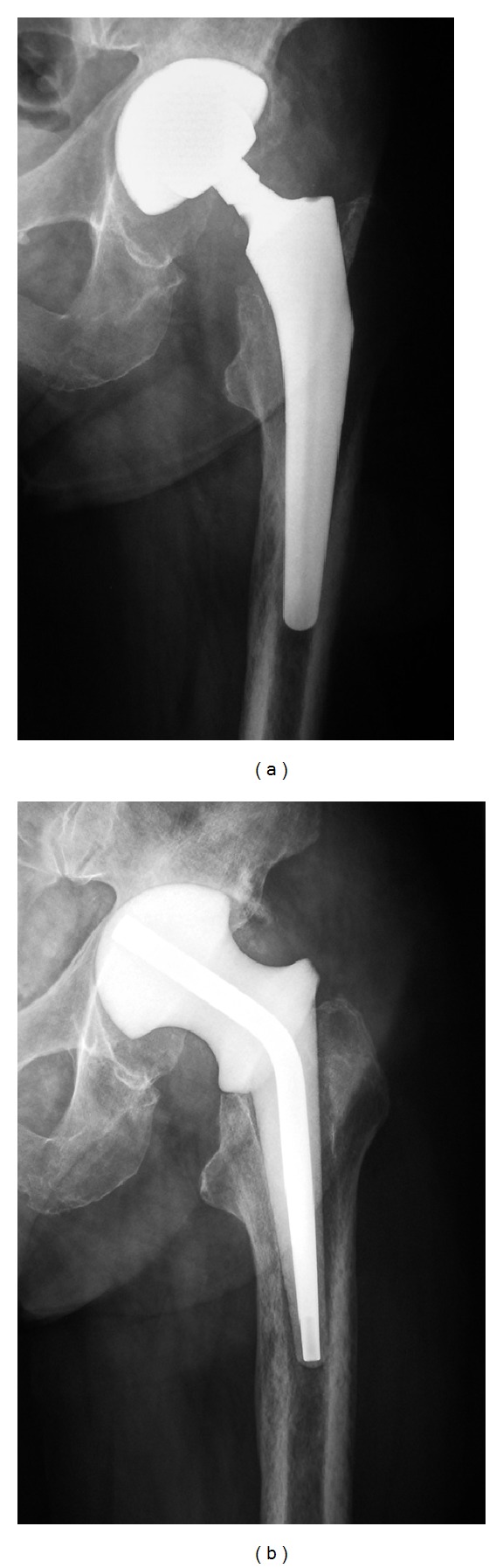
Preoperative X-ray of a total hip arthroplasty complicated by chronic infection (a). Radiograph obtained after removal of the infected prosthesis and implantation of an industrially manufactured spacer (b).

**Figure 2:**
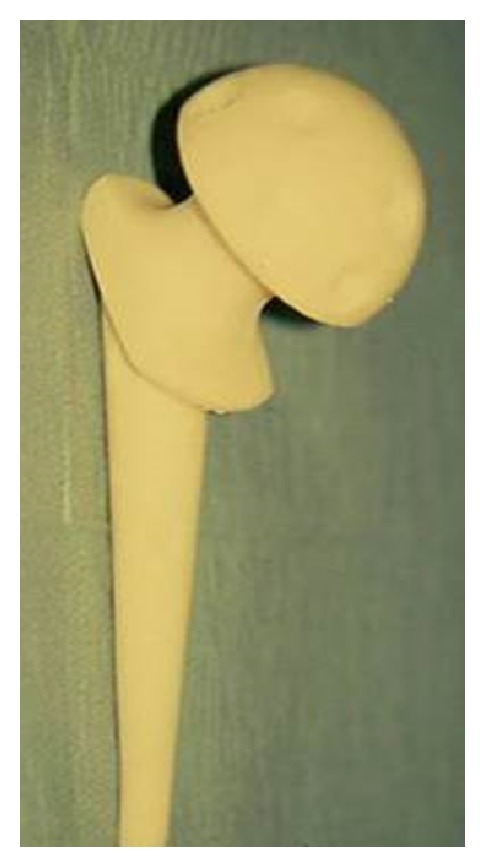
Image of the preformed gentamicin-loaded cement spacer after surgical addition of vancomycin using the “surface drill hole” technique.

Removal of the septic implant was followed by a thorough periprosthetic debridement and implantation of the spacer (Figures 1(a) and 1(b)). A vancomycin-loaded bone cement was prepared manually by mixing 40 g of powered cement PMMA polymer (Cemex, Tecres S.p.A., Sommacampagna, Verona, Italy) and 1 g of vancomycin (Vancocin, Eli Lilly, Milan, Italy). Finally, 35 mL of liquid MMA monomer was added and carefully mixed with a spatula [11, 19]. Vancomycin addition to hip spacers was obtained by filling with the cement mixture 17-18 holes (10–12 mm diameter, 2-3 mm depth) which were drilled in the surface of the Spacer-G immediately before implantation (Figure 2). Each device received 6-7 g of cement, corresponding to 150–170 mg of vancomycin, respectively. Vancomycin (Vancocin, 1 g, twice daily) was also administered intravenously to 1 patient as control case. 

Two- to 3-week standard parenteral antibiotics administration (cefazolin, Cefamezin, Pfizer Italia, Roma, Italy; 1 g four times a day; i.v.) was given to the remaining patients, followed by oral therapy, according to infectious disease consultant, for an overall treatment of 6 weeks. Outpatients clinical evaluation was arranged monthly, including laboratory tests (WBC, ESR, and CRP) and radiographic examination (anteroposterior and lateral views). Due to an immediate pain relief after surgery, a standard physiotherapy regimen including continuous passive motion was carried out. Partial to total weight bearing on the operated leg using two crutches was allowed until reimplantation.

In all the cases, eradication of infection was obtained, and the second surgical step, including the removal of the spacer and the application of a new THA, was performed successfully when patient's laboratory indices became normal and when bone scintigraphy with labeled leukocytes was negative for infection. In the postoperative period, parenteral antibiotic treatment was administered for 6 weeks in all the patients according to the pathogen identification or with broad-spectrum antibiotics in case of lack identification (2 patients) starting from day 3rd with the exception of the patients control case which started preoperatively.

Fluids drainage and serum samples to 1, 4, and 24 hours after the first surgical step were collected in all cases. Concentrations of gentamicin and vancomycin were determined in parallel by Fluorescence Polarisation Immunoassay (TDx, Abbott). The lowest measurable level of drug concentration was defined as that which could be distinguished from 0 with 95% confidence; this was determined as 0.27 mg/L for G and 2.0 mg/L for V [11]. The antibacterial activity determination was also done on different orthopaedic strains isolates with differing degrees of resistance. Bacterial strains were multiresistant clinical isolates obtained from Intensive Care Unit in patients, kindly provided by the Microbiology Department of the local university (Table 1).

The MICs of gentamicin, vancomycin, and their combinations were determined using the broth microdilution technique as recommended by the CLSI (Clinical and Laboratory Standards Institute) guidelines [20]. Resistance of the staphylococcus strains was determined according to international standard methods [21]. Resistance to gentamicin was defined by MIC_90_ > 32 mg/L; gentamicin-intermediate resistance by MIC_90_ = 8.0 mg/L, and resistance to vancomycin by MIC_90_ > 4.0 mg/L for the strains tested. Synergy testing was performed in duplicate using the chequerboard method in microtiter plates with Mueller-Hinton Broth (MHB, Difco). Gentamicin and vancomycin were diluted in MHB and tested at different twofold concentrations (from 0.3 to 20.0 mg/L) against all strains (final inoculum 1 × 10^5^ CFU/mL). The fractional inhibitory concentration index (FICI) was calculated and interpreted for each strain [22]: the FICI was defined as synergistic if the values were <0.5, indifferent or additive if the values were from 0.5 to 4.0, and antagonistic if the values were >4.0 [23] (Table 1).

Twofold serial dilutions of patient drainages were prepared in microtiter plates using Mueller-Hinton Broth as diluent. The final volume was 0.1 mL in each well, and 0.01 mL of each strain from overnight cultures was added to each well, including a growth control well, without drainage; an absolute control (MHB only) was also provided. Microplates were incubated for at least 18 h at 37°C. Subcultures for the 99.9% bactericidal endpoints were performed in Brain Heart Agar. The drainage fluid bactericidal titer (DBT) is a measure of the drainage fluid killing capacity against the infecting organism; it was determined as the highest fluid dilution achieving 99.9% bacterial killing. The score 3, corresponding to a 1/8 dilution, was considered the lowest effective titer for orthopaedic infections [24, 25]. 

## 3. Results

The release of gentamicin from PMMA cement at the site of infection showed high local concentrations (range 15.0–90.0 mg/L) in the first few hours after spacer implantation. Gentamicin serum levels were invariably very low (<0.2–1.0 mg/L). The local administration of vancomycin (2.5%) produced high concentrations (ranging from 13.8 to 40.0 mg/L) at the implant site in the first hour. This behaviour persisted 4 and 24 hours after spacer implantation. The corresponding serum levels were below the threshold for systemic toxicity (<10 mg/L for gentamicin and <40 mg/L for vancomycin); however, vancomycin attained therapeutic concentrations after parenteral administration, but again below systemic toxicity limits.

The levels of each antibiotic in drainage fluids were all above the concentrations needed to inhibit susceptible bacteria, and their use in combination appears to be capable of exerting pronounced antimicrobial activity and also a synergistic effect against some multiresistant microorganisms.

The DBT score was high (above 3) in the first few hours after drug release (1/8 titer) for all strains tested; an effective titer was maintained for almost 24 hours. The same drainage fluid presented different inhibitory capacities against various multiresistant strains. For example, patient n. 1 exhibited good inhibitory activity (DBT = 6) against* E. coli*, *S. aureus*, and lower inhibitory activity (DBT = 4) against *P. aeruginosa*; patient n. 6 had good inhibitory activity (DBT = 7) against *S. epidermidis *Methicillin-resistant and lower inhibitory activity (DBT = 3-4) against *S. aureus*,* S. haemolyticus* (2 strains), and *S. epidermidis*. Patient n. 7 showed good inhibitory activity (DBT = 6) against *S. hominis*, *S. aureus* and lower inhibitory activity (DBT = 4) against *S. haemolyticus* (2 strains) and *S. epidermidis*. Moreover, the fluid maintained high activity against the Gram-negative strains *E. coli* and *P. aeruginosa* (Table 2). 

The fluid collected from patient who also received local and systemic vancomycin (control case) was inhibitory against the majority of tested strains and higher against *S. aureus* during the first 24 hours after implantation. 

Depending on the different antibiotic concentrations in the microtiter plates and microorganisms tested, the samples inhibitory activity was variable. DBT scores indicated good inhibitory activity after 24 hours when G and V in combination were present at adequate concentrations (in these conditions, ≥8 mg/L and ≥2 mg/L, resp.) and when the G : V ratio was at least 2 : 1. 

Pain relief after application of the spacer was obtained in all cases, and partial weight bearing with crutches was allowed. There were no general or local complications (dislocation, breakage, and loosening of the spacer). No adverse drug reaction (hypersensitivity, erythema, edema, etc.) attributable to gentamicin or vancomycin was reported after local and systemic drug administration.

## 4. Discussion 

Periprosthetic hip infection following THA is a serious problem, and different treatment options related to the type of infection are available. In two-stage revision procedure, temporary spacers made of antibiotic-loaded PMMA represent a viable option for a chronically infected THA, allowing local antibiotic delivery and maintaining soft tissue length, which facilitates reimplantation [10, 26].

Industrially preformed spacer has some advantages such as ease of use, high availability in sizes, and excellent acetabular bone quality at the time of revision [26]. With the use of this specific device, many authors have reported good eradication rate ranging from 80 to 93.3% [26–29]. Industrial production ensures procedure standardization eliminating the time necessary to intraoperative manufacturing [26]. However, spacer-related complications, such as dislocations and fractures, have been described as well, ranging from 3.3 to 17% [26–29].

In this study, the release of gentamicin and vancomycin in the first 24 hours after implantation of hip preformed spacers was evaluated. Gentamicin and vancomycin concentrations were very high and strongly bactericidal in suction drainage fluid samples one hour after spacer implantation and remained high for at least 24 hours. These results confirm the findings of Anagnostakos et al., who firstly reported high concentrations of antibiotics in drainage fluids in the first few days after implantation of beads or spacer [10]. In addition, we observed different inhibitory capacities exerted by the same drainage fluid against several multiresistant clinical isolates.

Gentamicin and vancomycin concentrations determined singly in drainage fluids in the first 24 hours were very high and stable but not inhibitory against multiresistant strains. However, gentamicin and vancomycin act sinergistically against several multiresistant staphylococcal strains, as shown by the FICI and the DBT scores. The therapeutic rationale for combining G and V depends on the susceptibility of the infecting pathogens, and vancomycin use should be limited to infections likely to be caused by more resistant Gram-positive bacteria, such as *S. epidermidis*, methicillin-resistant staphylococci, CoNS, or enterococci [30, 31]. Cefazolin, not dosed, could contribute to antimicrobial activity of drainage fluids; it is effective mainly against susceptible strains, *S. aureus* (9A28) and *S. aureus* (3A10), and ineffective against the multiresistant strains as confirmed recently [32].

In our patients, gentamicin and vancomycin serum levels were below the threshold for systemic toxicity, and no signs of nephrotoxicity or local cytotoxic effects were observed. These data confirm the safety aspects of local drug delivery and the good tolerability of systemic and local levels. A low frequency of adverse reactions has been reported with antibiotic-containing spacers [10], though damage to the kidney and increased mortality has also been reported. In a systematic review including 10 observational studies, Luu et al. [33] showed an average incidence of acute kidney injury of 4.8% using antibiotic spacer. Berend at al. [34] studied mortality rates associated with two-stage treatment of infected THA in 202 patients undergoing two-stage treatment for infection, including removal of all implants and foreign material with implantation of an antibiotic-loaded cement spacer in the first stage followed by intravenous culture-specific antibiotics for a minimum of 6 weeks. Fourteen patients (7%) died before reimplantation, and two were not candidates because of medical comorbidities. The 90-day mortality rate after the first-stage debridement was 4%.

## 5. Conclusions

The results of the present investigation provide data on the release of gentamicin and vancomycin from preformed antibiotic cement spacers in the first 24 hours after implantation, supporting the potential clinical efficacy of the gentamicin-vancomycin combination in two-stage management of infected THA. Preformed spacers loaded with G and V are a safe method of delivering high concentration of antibiotics to the infection site with low serum levels, achieving effective release kinetics. The use of industrially preformed spacers should be advantageous in terms of standardization of the device characteristics, uniform cement mix with antibiotics, and reproducible drug release.

## Figures and Tables

**Table 1:** *In vitro* activity of gentamicin and vancomycin in combination against multiresistant clinical isolates.

Vancomycin + gentamicin			
Strain	MIC (mg/L)		
	Vancomycin	Gentamicin	FICI
*S. aureus *	2.5	10	0.15
*S. epidermidis *	2.5	3750	1.00
*S. haemolyticus *	1.25	3750	1.00
*S. haemolyticus* Methicillin-resistant	1.25	3750	1.00
*S. epidermidis* Methicillin-resistant	2.5	58.6	0.50
*S. hominis* Methicillin-resistant	1.25	15	1.02
*E. coli *	156.25	5.0	0.25
*P. aeruginosa *	1250	5.0	0.12

**Table 2:** Bactericidal titer of drainage fluids collected from 7 patients within the first 24 hours of spacer implantation against multiresistant clinical isolates.

Strain	DBT													
	PT 1		PT 2		PT 3		PT 4		PT 5		PT 6		PT 7	
	1 h	24 h	1 h	24 h	1 h	24 h	1 h	24 h	1 h	24 h	1 h	24 h	1 h	24 h
*S. aureus *	6	9	9	9	6	5	5	5	3	2	3	4	6	4
*S. epidermidis *	3.3	6	5	3	4	4	4	4	0	1	4	4	5	3
*S. haemolyticus *	3.3	6	6	7	4	3	4	4	0	1	3	3	4	3
*S. haemolyticus* Methicillin-resistant	3	5	4	3	4	4	4	3	0	0	4	5	4	3
*S. epidermidis* Methicillin-resistant	3.3	6	4.3	2	4	3	4	4	0	0	7	7	4	3
*S. hominis* Methicillin-resistant	5	8	7	5	5	5	4	5	0	1	5	5	6	4
*E. coli *	6	9	5	6	4	4	4	5	2	1	5	3	6	4
*P. aeruginosa *	4	6	5	5	4	4	3	4	0	0	5	6	5	3
